# Role of Mitochondrial Protein Import in Age-Related Neurodegenerative and Cardiovascular Diseases

**DOI:** 10.3390/cells10123528

**Published:** 2021-12-14

**Authors:** Andrey Bogorodskiy, Ivan Okhrimenko, Dmitrii Burkatovskii, Philipp Jakobs, Ivan Maslov, Valentin Gordeliy, Norbert A. Dencher, Thomas Gensch, Wolfgang Voos, Joachim Altschmied, Judith Haendeler, Valentin Borshchevskiy

**Affiliations:** 1 Research Center for Molecular Mechanisms of Aging and Age-Related Diseases, Moscow Institute of Physics and Technology, 141701 Dolgoprudny, Russia; andrey.bogorodskiy@phystech.edu (A.B.); ivan.okhrimenko@phystech.edu (I.O.); burkatovskiy.ds@phystech.edu (D.B.); ivan.v.maslov@phystech.edu (I.M.); g.valentin@fz-juelich.de (V.G.); norbert.dencher@physbiochem.tu-darmstadt.de (N.A.D.); 2 Environmentally-Induced Cardiovascular Degeneration, Central Institute of Clinical Chemistry and Laboratory Medicine, Medical Faculty, University Hospital and Heinrich-Heine-University Düsseldorf, 40225 Düsseldorf, Germany; philipp.jakobs@hhu.de (P.J.); joalt001@uni-duesseldorf.de (J.A.); juhae001@hhu.de (J.H.); 3 Institute of Biological Information Processing (IBI-7: Structural Biochemistry), Forschungszentrum Jülich, 52428 Jülich, Germany; 4 JuStruct: Jülich Center for Structural Biology, Forschungszentrum Jülich, 52428 Jülich, Germany; 5 Institut de Biologie Structurale (IBS), Université Grenoble Alpes, CEA, CNRS, 38400 Grenoble, France; 6 Physical Biochemistry, Chemistry Department, Technical University of Darmstadt, 64289 Darmstadt, Germany; 7 Institute of Biological Information Processing (IBI-1: Molecular and Cellular Physiology), Forschungszentrum Jülich, 52428 Jülich, Germany; t.gensch@fz-juelich.de; 8 Institute of Biochemistry and Molecular Biology (IBMB), Faculty of Medicine, University of Bonn, 53113 Bonn, Germany; wolfgang.voos@uni-bonn.de; 9 IUF—Leibniz Research Institute for Environmental Medicine, 40225 Düsseldorf, Germany

**Keywords:** mitochondria, mitochondrial protein import, age-related diseases, Alzheimer’s disease, Parkinson’s disease, cardiovascular disease, TERT, cardiolipin

## Abstract

Mitochondria play a critical role in providing energy, maintaining cellular metabolism, and regulating cell survival and death. To carry out these crucial functions, mitochondria employ more than 1500 proteins, distributed between two membranes and two aqueous compartments. An extensive network of dedicated proteins is engaged in importing and sorting these nuclear-encoded proteins into their designated mitochondrial compartments. Defects in this fundamental system are related to a variety of pathologies, particularly engaging the most energy-demanding tissues. In this review, we summarize the state-of-the-art knowledge about the mitochondrial protein import machinery and describe the known interrelation of its failure with age-related neurodegenerative and cardiovascular diseases.

## 1. Introduction

In general, mitochondria are known as the powerhouses of cells since their major function is to produce ATP as an energy source. Besides that, mitochondria are required for the regulation of calcium homeostasis, lipid and amino acid metabolism, and biosynthesis of heme and iron-sulfur complexes. Over the last years, it has become evident that mitochondria are signaling organelles, which regulate processes like apoptotic cell death as well as nuclear transcription [1].

Mitochondrial functions decline with the aging process of the organism, as mitochondria are subjected to a variety of biochemical stress conditions. Those lead to the accumulation of damaged molecules that directly or indirectly interfere with the regular biochemical processes occurring in mitochondria. Most prominent in this context are oxidative stress reactions caused by the accumulation of reactive oxygen species (ROS). ROS are generated as byproducts during the metabolic reactions of the OXPHOS process, in addition to detrimental environmental impacts [2]. Mitochondrial biogenesis capacity declines at older age, in particular due to the accumulation of mutations in the mitochondrial and nuclear genome. These mutations lead to the generation of defective or aberrant enzymatic components of the mitochondrial structure and metabolism, resulting in mitochondrial and eventually cellular dysfunction. This general problem is exacerbated by a relatively error-prone mitochondrial genome replication and inefficient repair processes [3].

As cellular survival depends on the maintenance of protein function, also called proteostasis, an efficient protein biogenesis process is a prerequisite to providing the required proteins for all cellular functions. Due to the dual genetic origin of mitochondrial proteins, a portion is encoded in the mitochondria, but most are nuclear-encoded; efficient protein biosynthesis and subsequent import into the organelle are essential for mitochondrial function. In addition to the transport processes into the organelle, mitochondrial polypeptides—irrespective of their source—need to undergo proper folding and assembly into active enzymes or enzyme complexes. Thus, the biogenesis of mitochondrial proteins represents a very complex process that is also prone to errors and defects correlating with diseases. Of note, not only the proteins in the inner (IMM) and outer mitochondrial membranes (OMM) are required for proper complex assembly, but also the phospholipid composition of the mitochondrial membranes plays a critical role. For example, it has been demonstrated that the lipid composition changes with age; in particular, the content of the mitochondria-specific lipid cardiolipin declines, which may play a central role in age-related diseases [4]. Age-related accumulation of cholesterol in mitochondria is a proposed trigger of Alzheimer’s disease (AD) [5,6].

Stress- or age-related damage of individual proteins or even the full organelle is counteracted by an array of different protective processes. On the protein side, a variety of chaperone and protease enzymes help to refold or remove damaged polypeptides before they accumulate and induce malfunction. To deal with unfolded polypeptides, mitochondria contain the full set of Hsp70- and Hsp60-type chaperones, similar to their bacterial ancestors [7]. In addition, each mitochondrial subcompartment, including the membranes, contains protease enzymes that specifically digest polypeptides that could not be refolded or assembled into their respective enzyme complexes [8]. Failure of this protein quality control (PQC) system contributes to many human diseases [9]. Specific signaling processes from mitochondria to the nucleus, summarized under the expression “unfolded protein response (mtUPR)”, increase the mitochondrial PQC capacity by enhancing the expression of the respective chaperones and proteases. If these protective processes fail, defective mitochondria themselves seem to be removed by a regulated and specific form of autophagy, named mitophagy [10].

As mitochondrial functions and quality control are largely dependent on the import of proteins produced in the cytoplasm, disruptions in import create cascading effects in mitochondria leading to diminished metabolic function, increased production of ROS, and failures in regulated cell death response [11]. Mitochondrial dysfunction is increased particularly with older age. For several diseases, most importantly affecting organs that particularly depend on mitochondria, namely the brain and the heart, mitochondrial dysfunction has been broadly described [1,12,13,14,15].

Not surprisingly, a number of recent reviews describe different aspects of mitochondrial function and structure, in particular mitochondrial machineries for protein import and assembly [16]. Considerably fewer reviews can be found on the connection between mitochondrial protein import and aging-related diseases [17].

This review provides an up-to-date overview of mitochondria translocation and sorting machinery and highlights the connection between its malfunction and diseases. We discuss the role of defective mitochondrial protein import in the two most common neurodegenerative diseases (Parkinson’s and Alzheimer’s diseases) and cardiovascular diseases (CVD), focusing on cardiolipin and the unique protective role of mitochondrial telomerase reverse transcriptase (TERT).

## 2. Mitochondrial Protein Import Machinery

Nuclear-encoded mitochondrial proteins typically contain signaling sequences that are recognized in the cytosol by the mitochondrial protein import machinery and transported into mitochondria in an ATP-dependent manner (for review, see [16]). Mitochondrial targeting sequences can either be *N*-terminal (e.g., subunit 8 of cytochrome c oxidase), internal (e.g., ADP/ATP carrier), or *C*-terminal (e.g., VDAC) [18,19,20,21]). *N*-terminal presequences are the most frequent and can be recognized by mitochondria import machinery either during or after translation [22]. After import, around 70% of presequences are cleaved by mitochondrial processing peptidases (MPP) [23]. Internal and *C*-terminal mitochondrial targeting sequences can only be recognized after protein translation is complete. Mitochondrial proteins employing internal or *C*-terminal targeting sequences are frequently hydrophobic and require assistance from chaperones such as Hsp70/90 to prevent misfolding and aggregation in the cytoplasm.

**Figure 1:**
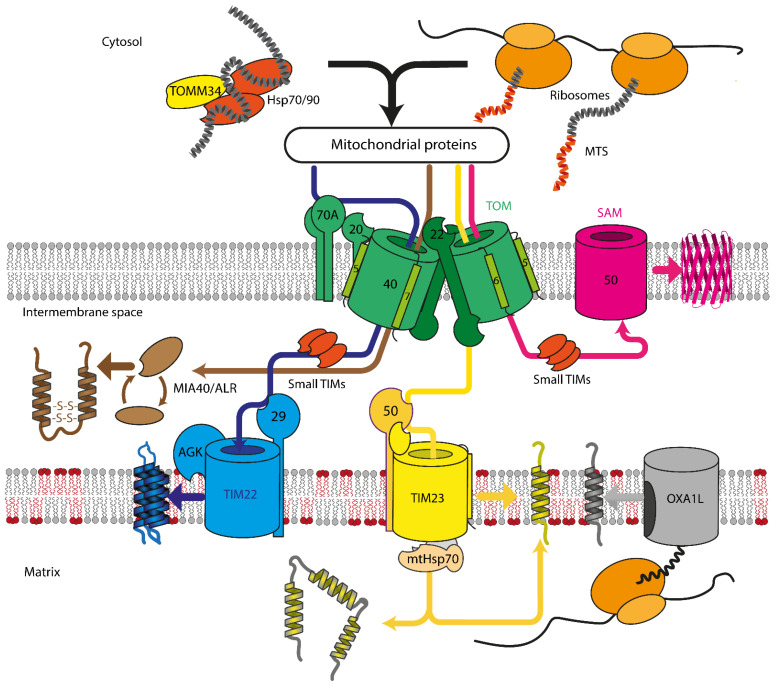
Overview of mitochondrial protein import in mammalian cells. Proteins are recognized by TOMM70A/TOMM20/TOMM22 and are imported either co- or post-translationally through the TOM complex, containing TOMM40, TOMM22, TOMM5, TOMM6, and TOMM7. Hydrophobic proteins employ Hsp70/90 complex with participation of TOMM34 to prevent misfolding in the cytoplasm. Inside the intermembrane space, depending on the nature and destination of the precursor protein, proteins are delivered to different compartments. β-barrels of the outer membrane are inserted into the outer mitochondrial membrane by SAM complex. Intermembrane space proteins with cysteine motifs are oxidized to the mature form by the MIA40/ALR system. Metabolite carriers are inserted into IMM by TIM22 complex, composed of TIMM22, TIMM29, and acylglycerol kinase (AGK). Other IMM and matrix proteins are inserted/transported by TIM23 complex. Primary TIMM23 pore is associated with TIMM50 (recognizes signals and interacts with TOM complex), TIMM44 with associated mtHsp70 (forming presequence-associated motor helping matrix protein transfer), or TIMM21 for protein release into IMM. Mitochondrial-encoded proteins are inserted into the IMM by OXA1L insertase from the matrix side.

Mitochondrial protein recognition occurs at the translocase of the outer membrane (TOM) complex. The TOM component TOMM20 acts as a direct receptor for *N*-terminal signal sequences, while TOMM70 seems to be dedicated to the recognition of hydrophobic proteins with internal signal sequences. These hydrophobic proteins reach the mitochondrial surface, often bound by cytosolic chaperones that also can interact with TOMM70 [16]. After passing through pore formed by the TOM complex, proteins are sorted between TIM22, TIM23, SAM, or MIA machinery, depending on the protein destination and fold (Figure 1). Insertase OXA1L inserts mitochondria encoded proteins into IMM. In yeast, mitochondrial insertase Oxa1p additionally assists in inserting multi α-helical nuclear-encoded proteins, but the same behavior for human OXA1L was not yet verified [24,25].

In addition to signaling sequences, mitochondrial protein import efficiency is further enhanced by localization of mRNA encoding mitochondrial proteins near the OMM and its translation in the vicinity of the translocation machinery [26,27,28], thus bypassing the need for intermediate chaperone-mediated transport. The observed mRNA localization near OMM is guided either by the interaction of protein nascent chains with TOM complex or by the interaction between 3′ mRNA end, specific RNA-binding proteins (RBPs), and OMM proteins. In human cells, RBPs are only incompletely described, but some proteins were found to co-localize with mRNA on the OMM surface, e.g., CluH and PUM [29,30]. Recent advances in proximity labeling identified several new RBPs [31] on the OMM surface, including SYNJ2BP, which persists on the OMM surface after disruption of protein synthesis and binds several mRNAs of OXPHOS proteins. RBPs’ participation in mitochondrial protein transport presents another potential avenue of protein import disruption caused by mutations/aging/environmental factors.

### 2.1. Translocase of Outer Mitochondrial Membrane

The TOM complex is the main entry gate for the mitochondrial proteins, consisting of the proteins TOMM40, TOMM22, TOMM20, TOMM70A, TOMM5, TOMM6, and TOMM7. TOMM40 forms the main transmembrane pore of the TOM complex, while the rest of TOMM proteins help in signal recognition, complex stabilization, and assembly.

Both dimeric and trimeric structures of the human TOM complex were observed using CryoEM and chemical cross-linking [32,33]. In dimeric form, twin tilted β-barrels of TOMM40 are flanked by twin TOMM22 proteins, and each TOMM40 is surrounded by singular TOMM5,6,7 proteins, which help with complex stabilization [32,34] (Figure 1). TOMM22, TOMM6, and TOMM40 have exposed negatively charged surface patches which serve as recognition sites for positively charged signaling sequences of mitochondrial proteins. TOM complex may also form trimers, which are likely required for lateral release of protein into the OMM [33], but otherwise, trimer formation is relatively poorly understood.

TOMM70A, weakly attached to the TOM complex, recognizes hydrophobic cytosolic proteins with internal mitochondrial targeting sequences. Transport of such proteins in the cytosol is frequently assisted by the Hsp70/Hsp90 chaperone complex and, possibly, by co-chaperone TOMM34. [35,36]. TOMM20, also weakly associated with TOM complex, is a receptor for mitochondrial protein precursors with *N*-terminal targeting signals. TOMM20 additionally assists TOMM70A during protein translocation [35].

After being imported through the TOM complex, proteins are sorted by downstream complexes. Sorting and assembly machinery (SAM) complex inserts β-barrels such as porins into OMM. Mitochondrial intermembrane space assembly (MIA) assists in the maturation of IMS proteins with cysteine motifs. Translocase of inner mitochondrial membrane (TIM) 22 and 23 complexes transfer protein to IMM and matrix, with TIM22 used exclusively for IMM metabolite carrier proteins, while TIM23 transports the rest of the proteins. More details of these processes are given in the following sections of this chapter.

### 2.2. Sorting and Assembly Machinery Component

The SAM complex governs the assembly of β-barrel proteins (such as porins, TOMM40, and SAMM50 itself) of the outer membrane after their precursors were translocated through the TOM complex. In yeast and, likely, in humans, β-barrel proteins pass through TOM complex employing internal or *C*-terminal β-hairpin as a signaling sequence [21]. After emerging from the TOM complex, small Tim chaperones bind to the precursor proteins to prevent their aggregation; then, proteins are passed to the SAM complex that embeds β-barrel into the OMM from the IMS side. Partner proteins of SAMM50 are not well defined in humans [37]. Evidence exists that metaxins partner with SAMM50, e.g., metaxin-1 forms a complex with SAMM50 [38,39,40]. However, only a small portion of metaxin-1 is associated with SAMM50, and a large portion exists in a separate complex with metaxin-2 [40].

### 2.3. Mitochondrial Intermembrane Space Assembly

The import of small IMS proteins with multiple cysteine residues occurs via the mitochondrial intermembrane space assembly (MIA), which captures imported proteins in the IMS via the thiol-disulfide exchange. MIA substrates possess characteristic cysteine motifs CX_3_C and CX_9_C (where X are amino acids), which are oxidized to form mature forms of proteins in the IMS. MIA consists of two proteins: Mia40 (alternatively named CHCHD4—Coiled-Coil-Helix-Coiled-Coil-Helix Domain Containing 4) and ALR (augmenter of liver regeneration). MIA substrates are kept in reduced form during translocation through the outer membrane and are oxidized by Mia40 in IMS. Mia40 is then reoxidized by the ALR protein, which in turn is interacting with cytochrome c [41]. Similar to the substrates import, Mia40 requires its own entrapment by oxidation of twin CX_9_X motifs. Oxidation-driven import of the IMS-targeted proteins is much slower than the import of presequence targeted proteins and is strongly dependent on the availability of MIA40 [42]. After oxidation, mature proteins of the IMS are released.

### 2.4. Translocase of Inner Mitochondrial Membrane

The inner mitochondrial membrane contains two distinct translocation complexes: TIM22 and TIM23. TIM22 is used for the import of some IMM proteins containing internal signaling sequences, while TIM23 is used for the remaining IMM proteins and all the matrix proteins. TIM23 is much more abundant and possesses the ability to change the mode of transport depending on the attached subunits.

#### 2.4.1. TIM23

The translocase of the inner mitochondrial membrane 23 (TIM23) complex sorts proteins to the matrix or IMM. The main components of the TIM23 complex are the proteins TIMM23 (main pore), TIMM50, TIMM17, and TIMM44 [43]. TIMM17 has two variants, TIMM17A and TIMM17B, with the latter being more abundant [44]. TIMM50 recognizes presequence-containing proteins on the TOM complex IMS side and passes them into the TIM23 complex [45]. TIMM44 regulates the attachment of mtHsp70 (mortalin) to the TIM23 complex from the matrix side. mtHsp70 binds to the precursor proteins and uses ATP hydrolysis to pull them inside. mtHsp70 co-chaperones such as MAGMAS, MCJ, and DNAJC19 also interact with TIM23 complex and modulate the ATPase activity of mtHsp70 on the matrix side of the IMM [45]. Together, TIMM44, mtHsp70, MAGMAS, and DNAJC19 form a presequence-associated motor (PAM), which facilitates ATP-dependent protein import into the matrix. Inside the matrix, MPP remove presequences, and the mature form of imported protein is released. Single α-helical proteins are released from TIM23 directly into the IMM with the help of TIMM21 and ROMO1, with TIMM21 substituting PAM assembly in that case.

#### 2.4.2. TIM22

TIM22 complex inserts into IMM hydrophobic α-helical proteins containing multiple internal targeting sequences, which include metabolite carriers and subunits TIMM17, TIMM23, and TIMM22 of translocases [46]. TIMM22 protein is laterally exposed into the membrane cavity and, together with TIMM29 and AGK, forms the TIM22 complex [47,48,49]. TIMM29 interacts with the TOM complex, further facilitating the transfer of proteins from the cytosol into IMM [50]. Hydrophobic α-helical proteins are likely delivered by hexamer complexes of TIMM9/TIMM10a and TIMM9/10a/10b, which were found directly attached to the TIM22 complex [48].

### 2.5. Mitochondrial Lipids

Essentially, the complete translocation and sorting machinery consist of membrane-bound or transmembrane complexes. Their assembly and functioning are influenced by the lipid composition of the mitochondrial membranes. Many mitochondrial lipids originate from the endoplasmic reticulum and Golgi complex, but some of the crucial lipids are synthesized directly in mitochondria [51,52]. In eukaryotes, cardiolipin is exclusively synthesized in the mitochondria, where it remains throughout the life of the cell.

Studies in yeast revealed that cardiolipin influences the import of proteins not only via the TIM complexes (for review, see [53]) but also via the TOM complexes [54]. Besides its regulatory function on complex assembly of the electron transport chain (ETC) [52] as well as its unique role as a proton trap at the membrane surface participating in oxidative phosphorylation and therefore in ATP synthesis [55], cardiolipin also mediates interactions between Tim23 and Tim50 in yeast [56]. Cardiolipin was also found between TOMM22 and TOMM40 in the CryoEM structure of the human TOM complex, but the role of this arrangement is yet to be determined [33]. Mitochondrial membranes also contain low amounts of cholesterol and are sensitive to changes in cholesterol level [57,58], which is, in turn, finely regulated by the mitochondrial protein import machinery [59].

## 3. Neurodegenerative Diseases

Neurodegenerative diseases (ND) encompass conditions of cognitive impairment accompanied by changes in the behavior and functioning of the organism. Common for ND is the progressive death of nerve cells (neurodegeneration) in many cases related to mitochondria malfunction [60]. There is a bulk of evidence that malfunction of the mitochondrial protein import system causes ND or is associated with them. In this chapter, we review the evidence of the interrelation of mitochondrial protein import machinery with Parkinson’s and Alzheimer’s diseases.

### 3.1. Parkinson’s Disease

Parkinson’s disease (PD) is an ND that mostly affects the elderly population, with a prevalence of 2–3% for people above 65 years. PD patients suffer from bradykinesia, rigidity, rest tremor, sleeping and mood disorders, cognitive impairment, autonomic dysfunction, as well as sensory symptoms and pain (reviewed in [61]). The loss of dopaminergic (DA) neurons in the *substantia nigra* and the accumulation of α-synuclein (ASYN) are considered the main hallmarks of PD. It is likely that DA neurons suffer most from PD because of their high bioenergetic demands and axonal arborization [62]. This confirms that the mitochondria, being the cell powerhouses, play a key role in PD pathogenesis.

**Figure 2:**
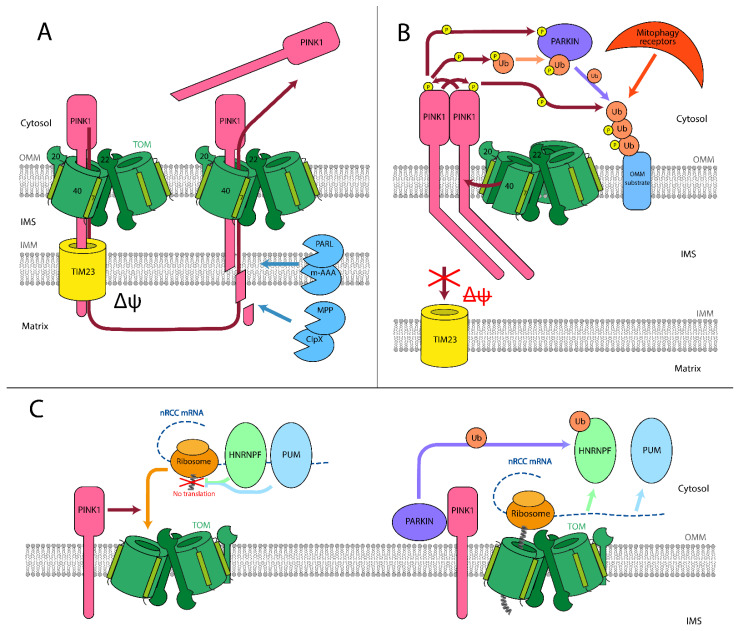
Role of mitochondrial protein import in Parkinson’s disease. (**A**) PINK1/PARKIN mitochondrial quality control model. In healthy mitochondria, PINK1 is transported through both TOM and TIM complexes, utilizing the IMM electrostatic potential. Afterward, it is cleaved inside the matrix and on the IMM by mitochondrial proteases. The remaining part of PINK1 is dislocated into the cytosol, where it is degraded. This process allows keeping PINK1 levels on the OMM negligible. (**B**) In unhealthy mitochondria, PINK1 is unable to be transported through the TIM complex. It is ejected from TOMM40 laterally into the OMM, and the ejection is facilitated by TOMM7. PINK1 remains in connection with TOMM20. In that state, it accumulates on the OMM and starts forming homodimers within an HMW PINK1 complex. In dimeric form, PINK1 phosphorylates its dimeric partner and starts to phosphorylate PARKIN and ubiquitin. Phosphorylated PARKIN and ubiquitin form complex and ubiquitinate targets on the OMM. PINK1 phosphorylates ubiquitin on such targets, which attracts mitophagy receptors, starting the mitophagy process. (**C**) Proposed *nRCC* translation regulation mechanism. While the *nRCC mRNA* is in the cytosol and away from the mitochondria, PUM and HNRNPF are attached to it and serve as translation repressors. PINK1 attracts mRCC mRNA to the TOM complex on the outer mitochondrial membrane. Then, PARKIN removes the translation repressors HNRNPF and PUM from the mRNA. For HNRNPF protein, this process involves ubiquitination by PARKIN. When the repressors are released, co-translational synthesis through the TOM complex starts.

PD can be initially caused by monogenic mutations (see review [63]), as well as harmful environmental factors and aging. Among the genes in which mutations have been identified that cause early-onset PD, *PINK1* and *PRKN* have been directly linked to mitochondria. They encode a mitochondrial protein, the PTEN induced kinase 1 (PINK1), and a cytosolic E3 ubiquitin ligase (PARKIN/PRKN), that is recruited to mitochondria under pathological circumstances. In healthy cells, these proteins act together as a sensor for mitochondrial quality and contribute to the maintenance of mitochondrial function [10] (Figure 2A). The *N*-terminal mitochondrial targeting sequence in PINK1 is partially transported through TOM and TIM23 complexes and cleaved by mitochondrial peptidases (MPP, PARL, m-AAA, and ClpXP) in the matrix [64]. The truncated PINK1 detaches from the mitochondrial membranes and gets degraded in the cytosol [65]. In stressed mitochondria with disturbed membrane potential, PINK1 cannot pass through TIM23. Instead, it accumulates at the OMM in the full-length form and forms a high molecular weight PINK1 complex (HMW PINK1 complex) that contains TOM complex proteins and a PINK1 dimer [66]. Although the mechanistic details and the exact role of the PINK1 complex on potential-deficient mitochondria is still not entirely clarified, it is well established that the accumulated full-length PINK1 recruits the cytosolic PARKIN to the mitochondrial surface. The process involves PINK1 phosphorylation and ubiquitin phosphorylation, which in turn activates PARKIN ligase activity [67]. Activated PARKIN ubiquitinates substrates on the OMM, which are afterward phosphorylated by PINK1. This attracts signaling proteins and induces autophagosome formation, thus eliminating the dysfunctional mitochondria [68] (Figure 2B).

The detailed composition of this HMW PINK1 complex is still not identified and is of interest in the context of mitochondrial transport. At least three TOM complex proteins (TOMM20, TOMM22, and TOMM40) have been commonly identified as HMW PINK1 complex components [69]. Additionally, TOMM7 was shown to be essential for the stabilization of PINK1 on OMM of stressed mitochondria [70]. The exact position of PINK1 in the complex is a matter of debate. Crosslinking experiments suggest that the accumulated PINK1 is associated with TOMM20 but not with TOMM40 in depolarized mitochondria [71]. Therefore, it is reasonable to expect that PINK1 is laterally released into the OMM. The number of TOMM40 components within an HMW PINK1 complex is also not determined yet. Recent cryo-EM structures of dimeric and trimeric human TOM complexes suggest that only trimeric TOMM40 can open laterally into the OMM and thus release PINK1 [33].

Taken together, the PINK1/PARKIN-dependent signaling pathway seems to play an important role in the removal of damaged mitochondria under stress conditions. However, most experiments analyzing PINK1 function used a treatment with uncoupling chemicals like CCCP, resulting in a complete loss of the inner membrane potential in all mitochondria within a cell—a situation that certainly does not reflect the situation in living organisms. Hence, it remains to be established what role the PINK1/PARKIN-induced mitophagy plays in the emergence of the Parkinson pathology in human patients.

Emerging evidence suggests that PINK1 and PARKIN may participate in the mitochondrial translation of nuclear-encoded respiratory chain complexes (nRCC) (Figure 2C) near the mitochondrial surface. While the *nRCC mRNA* is not located near the mitochondria, translation of *nRCC mRNA* is repressed by proteins PUMILIO (PUM) and HNRNPF. PINK1 accumulates on the OMM of mitochondria with impaired IMM potential, binds to TOM complex, attracts *nRCC* mRNA to the OMM, and facilitates association of the nascent chain with the TOM complex. PARKIN acts downstream of PINK1, ubiquitinating HNRNPF, thus displacing it from the *nRCC mRNA*. PINK1 and PARKIN are also associated with the displacement of other *nRCC mRNA* translation repressors: PUM, GW182, DCP1, and POP2 [72]. Interestingly, the described mechanism relies on PINK1 being integrated into the mitochondrial membrane, which, as mentioned before, is the first step of mitophagy. Therefore, *nRCC* synthesis activation may serve as the first immediate step for rescuing the damaged mitochondria.

Mutations in PINK1/PARKIN have been established as risk factors in hereditary forms of PD (see [63,73] for review). For some of these mutations, their mechanism of action has been studied. PINK1 mutations have been shown to affect its stability (I368N [74], L347P [75]), recruitment to mitochondria (S402A [66]), autophosphorylation (G309D, L347P [75], G411S [76]), ubiquitin phosphorylation (G411S [76]), and dimerization (Q456X [76]). Meanwhile, PARKIN mutations are known to cause its aggregation (C289G, C418R [77]) and loss of E3 activity (M434K, C441R [78]). All of these mutations interfere with the PINK1/PARKIN MQC and/or *nRCC* mRNA regulation system and presumably result in PD.

In addition to the more indirect involvement of the import system in the PINK1/PARKIN signaling pathway, an additional—more direct—pathological event has been proposed to take place during PD. Accumulation of mutant forms of α-synuclein (ASYN), another genetic factor in PD, as aggregated proteins in so-called Lewy bodies is considered a major hallmark of PD pathology. Some experiments directly link ASYN-based pathological pathways to defects in mitochondrial protein import. The expression of certain pathological forms of ASYN has been revealed to impair mitochondrial import of proteins [79]. However, the mechanism of this impairment remains elusive. TOMM40 levels have been shown to decrease in cells overexpressing ASYN [80], although the exact mechanism is not defined, as this decrease might be caused indirectly by mitochondrial damage. There is an experimentally supported theory that oligomeric, dopamine-modified, or S129E mutant ASYN might bind to the mitochondrial protein import receptor TOMM20, preventing its association with TOMM22, which would lead to a decrease in import through the TOM complex [79]. The transport of ASYN itself into the mitochondria is also debated. An earlier report proposed that ASYN is imported through the TOM machinery [81], although more recent data advocate against that, as no interaction with TOMM40 was detected [79]. Recent experiments point at a potential interaction of ASYN with the voltage-dependent anion channel (VDAC), a major pore protein in the OMM, suggesting that ASYN might be transported into mitochondria through it [82,83]. However, although the normally cytosolic ASYN may affect the pore properties of VDAC, a direct transport through the pore has not been demonstrated convincingly yet.

### 3.2. Alzheimer’s Disease

Alzheimer’s disease (AD) is the most common age-related form of ND (60–80% of all known dementia). AD occurs in only 3% of cases at the age of 70, but in more than 30% of cases at the age of 90 [84]. Despite the tremendous progress in science and medicine, the causes and mechanisms associated with this pathology remain unclear more than 100 years after the disease was first described [85].

Various mechanisms have been proposed for the involvement of Aβ peptides in AD. For a long time, the focus of research and treatment of Alzheimer’s disease has been on extracellular plaques consisting of Aβ peptides. According to the “amyloid hypothesis”, aggregates of amyloid fibrils that settle outside the neurons in dense formations (senile or neuritic plaques) are the causative agent of AD [86]. It is hypothesized that the intracellular Aβ peptides in human neurons are involved in AD before the formation of extracellular plaques [87,88,89].

As it is well established that mitochondria function declines with age, numerous attempts were made to elucidate a connection between mitochondrial dysfunction and AD [90]. Recently the hypothesis of the “mitochondrial cascade” emerged [91], which proposed a causative role of impaired mitochondrial function as a factor in the etiology of AD. The “mitochondrial cascade hypothesis” suggests mitochondria as a trigger and target for Aβ-mediated intracellular dysfunction and damage in the early stages of AD. The exposure to monomeric and oligomeric Aβ peptides (Aβ) negatively affects mitochondrial function, causing an increase in free radical (particularly ROS) stress and damage to the mitochondrial genome, thereby disrupting the normal functioning of neurons [92,93,94].

Additionally, Aβ directly and indirectly affects the mitochondrial protein import during AD. The clogging of TIM/TOM [95,96,97] or mitochondrial preprotein aggregation [98] lowers the import of mitochondrial proteins coded in the nucleus, particularly the subunits of the complexes of the oxidative phosphorylation machinery, which, in turn, affects the mitochondrial membrane potential (MMP, Δψ) [99]. Lowering the Δψ impairs protein import into mitochondria driven by electric field [100] and lowers the amount of ATP produced by mitochondria [101,102], but ATP, particularly, is needed for TIM to force the import protein polypeptide chain movement [103]. The positive feedback lets tiny deviations in protein import caused by prolonged Aβ action escalate and become important for the whole organism [104]. This is directly related to the importance of mitochondria function and constant high-intensity metabolism, which they drive [105,106,107].

**Figure 3:**
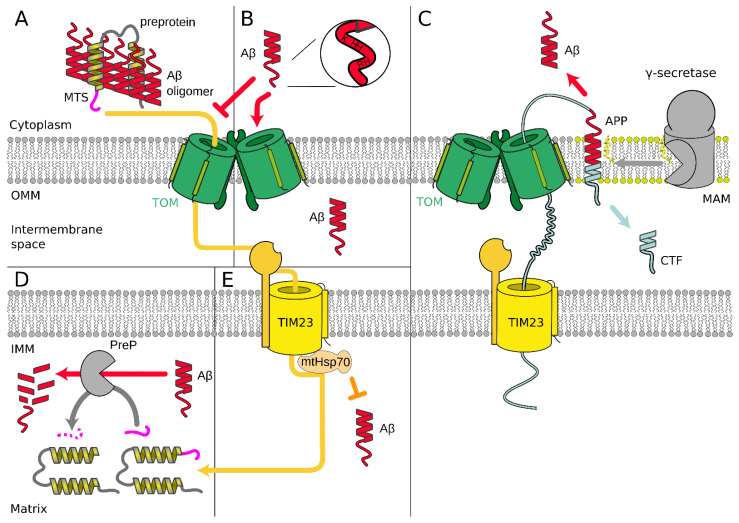
(**A**) Aβ peptides form cytosolic Aβ–preprotein coaggregates that inhibit the initial steps of the mitochondrial preprotein transfer through TIM/TOM complex [98]. (**B**) Positively charged amino acid residues of Aβ, localized closely at the *N*-terminal cation binding domain [115], are possibly sufficient for recognition by the TOM complex. Aβ species possibly could be imported into the mitochondria through the TOM complex by the same mechanism as proteins with the signal peptide of mitochondrial localization [116]. (**C**) Amyloid beta precursor protein (APP) in mitochondria-associated membranes (MAM) [108] is degraded by mitochondrial γ-secretase [112], producing Aβ [96,108,111]. APP possibly blocks TIM/TOM complex by arresting translocation [95,96,97]. (**D**) Mitochondrial presequence peptidase (PreP) is able to degrade Aβ [118]. Aβ possibly inhibits PreP function of degrading presequences and processing protein intermediates after translocation through TIM/TOM complex [119]. (**E**) Overexpression of mortalin (mtHsp70) suppresses Aβ-mediated mitochondrial fragmentation and cell death [124].

Amyloid beta precursor protein (APP) is localized in mitochondria-associated membranes (MAM; a special part of the endoplasmatic reticulum) [108]. The sequential proteolysis of APP by membrane secretase enzymes produces an Aβ peptide. APP fragment of amino acid residues number from 672 to 713 corresponds to Aβ_1-42_ peptide and 672–711 to Aβ_1-40_ [99]. Aβ peptides are continuously produced throughout life in a healthy brain, and an increase in either total Aβ levels or Aβ_1-42_/Aβ_1-40_ ratio is associated with a late onset of AD pathogenesis [109]. Some experiments showed a noticeable portion of APP in OMM [96,110,111]. Additionally, it was reported that APP is degraded in MAM and OMM by mitochondrial γ-secretase [112], producing the *C*-terminal fragment (CTF or C99 [109]) on the cytoplasmic side and probably Aβ [96,108,111]. Based on these few experiments, it was proposed that APP blocks TIM/TOM complex arresting translocation [95,96,97] (Figure 3C).

Aβ peptide indirectly affects the initial steps of the mitochondrial preprotein transfer through TIM/TOM complex by the formation of Aβ–preprotein cytosolic coaggregates outside mitochondria [98]. Importantly, Aβ_1–42_ induced 10 times greater effect than Aβ_1–40_. This correlates with the properties of Aβ_1–42_, which is more hydrophobic and has a greater tendency to form aggregates and amyloidogenic fibrils than Aβ_1–40_. However, Aβ_1–42_ and Aβ_1–40_ peptides did not affect the structure of preprotein translocase complexes and interacted only weakly with mitochondria without the influence on the inner membrane potential [98] (Figure 3A). These findings obtained for human cells are supported by similar data for *S. cerevisiae* and *C. elegans*. In the latter case, cytosolic aggregation of mitochondrial preproteins was observed, which are known to be downregulated in AD, and the aggregation of ASYN and Aβ was enhanced because of mitochondrial protein import dysfunction [113].

Relatively high (but sub-lethal) concentrations of oligomerized Aβ species inhibited the import of mtGFP with *N*-terminal mitochondrial targeting sequence (MTS) [21]. Notably, high concentrations of Aβ led to the decrease of the MMP, increase in ROS concentration, and altered mitochondrial morphology only after a long period of treatment, after the decline of import of two endogenous nuclear-encoded mitochondrial proteins, mortalin/mtHsp70 and Tom20 [114].

It was reported that Aβ_1–40_ and Aβ_1–42_ are imported in vitro into the mitochondria through the TOM complex by the same mechanism as mitochondria-targeted proteins (Figure 3B). Positively charged amino acid residues of Aβ, localized closely at the *N*-terminal cation binding domain [115], are possibly sufficient for recognition by the TOM complex. As the import was insensitive to valinomycin, it was concluded that Δψ is not necessary for the Aβ import, unlike other matrix proteins [116]. Unfortunately, no time-course experiments were performed, and conclusions are only based on co-sedimentation of Aβ and mitochondria [116,117].

Mitochondrial presequence peptidase (PreP) is responsible for degrading presequences and other short unstructured peptides just after translocation through TIM/TOM complex. PreP is also able to degrade Aβ_1–42_ and Aβ_1–40_ [118]. Furthermore, it was claimed that in *S. cerevisiae* and rat mitochondrial matrix, Aβ inhibits PreP degradation of presequences and nuclear-encoded mitochondrial proteins [119]. This, presumably, results in the accumulation of non-folded mitochondrial preproteins and processing intermediates, leading to the decrease of mitochondrial function [120] (Figure 3D). In a mouse model, the cognitive functions were partially restored by PreP overexpression [121]. However, this correlation does not directly connect PreP with the Aβ degradation in the mitochondrial matrix. Even more, this observation of Aβ inhibition of preprotein processing inside intact human mitochondria was experimentally excluded recently [90,98].

Mortalin, the mitochondrial chaperon Hsp70, is an essential part of the TIM23 complex [122,123]. The inhibition of mortalin induced mitochondrial fragmentation and increased Aβ-mediated cytotoxicity as well as mitochondrial dysfunction. Moreover, overexpression of mortalin suppressed Aβ-mediated mitochondrial fragmentation and cell death [124] (Figure 3E). These facts led to the conclusion that decreased functionality of mortalin influences protein import, particularly in AD (but also in PD and Huntington’s disease) [17]. It should be noted that as far as mortalin is involved in numerous processes in the cytoplasm and mitochondria [125], then the alteration of mortalin expression or functionality could exacerbate a systematic severe effect and influence on mitochondrial protein import could be indirect, especially because a relatively small portion of total cell mortalin is involved in TIM23 machinery.

The cholesterol amount in mitochondrial membranes is dependent on the activity of mitochondrial protein import complexes [59]. Mitochondrial membranes contain relatively low amounts of cholesterol, but these changes are important [57,58]. Cholesterol affects the Aβ maturation, directly influencing the activity of γ-secretase in MAM during proteolytic degradation of amyloid precursor protein (APP) [126,127,128]. Cholesterol also interacts with the membrane part of APP [129,130], affects the activity of β-secretase [131,132] and α-secretase [133], defining the sequential cleavage of APP either via the amyloidogenic or non-amyloidogenic pathway, respectively. The C99 fragment of APP is the precursor of Aβ peptides, also involved in Alzheimer’s disease. It regulates cellular cholesterol trafficking [134].

A high, non-physiological concentration of Aβ is often used in experiments to produce notable measurable effects. However, high concentration of any peptide could produce a severe effect on mitochondria, so the relation of obtained results to the mitochondrial protein import mechanisms in disease and healthy state is still not settled. The experimentally obtained decline of import of mitochondria targeted GFP of approximately 20% by treatment of predominantly aggregated Aβ_1-42_ at 10 μM concentration for 96 h [114] seems to be an illustration of this statement. Similar experiments with monomeric Aβ_1-40_ and Aβ_1-42_ at a concentration of 3.5 μM showed the formation of co-aggregates with mitochondrial-targeted proteins [98]. Therefore, the aggregation state of Aβ should always be taken into account during basic research because monomers, small oligomers, and fibrils of Aβ could produce different effects. We assume that a mixture of monomeric, oligomeric, and fibrillar species was applied in widely cited experiments in which the import of Aβ through TOM was studied [116]. Close to physiological concentration (100 nM) of Aβ was used, but without any pretreatment and no examination of the presence and amount of the oligomeric state of Aβ. At the same time, therapeutic approaches already exist, which target fibrils or monomers of Aβ [135], and even small oligomers [136,137].

## 4. Cardiovascular Diseases

Cardiovascular diseases (CVD) are the leading cause of death in the world. There are many factors known to increase the risk for CVD. The major modifiable risk factors include, e.g., unhealthy diet, physical inactivity, mental stress, and obesity. Despite the ongoing search for pharmaceuticals and improving healthcare, the risk of developing CVD is consistently high in developed countries. In Europe, more than 4 million people die every year from CVD. In 2016, cardiovascular complications, such as hypertension, atherosclerosis, pathological hypertrophy, ischemic heart disease, and myocardial infarction, resulted in economic costs in Europe amounting to a total of EUR 210 billion [138]. Thus, treatment of these complications has a tremendous impact. Therefore, an overall understanding of the underlying causes and mechanisms is required to find new treatments and improve those already established. It is indisputable that many cells of the cardiovascular system—including cardiomyocytes, smooth muscle cells, fibroblasts, and endothelial cells—rely on proper mitochondrial functionality. Moreover, accumulating evidence reveals novel links between mitochondrial dysfunction and CVD pathogenesis. Over the last years, mitochondrial dynamics, mitochondrial networks, and mitochondrial biogenesis have become of particular interest besides improper functioning of the respiratory chain and mitochondrial reactive oxygen species as potential new therapeutic routes in CVD. However, the mitochondrial protein import machinery has been greatly overlooked in most of the studies with respect to the cardiovascular system, although proteins have to be imported into the mitochondria for the proper functionality of these organelles [139]. Nevertheless, phenotyping of mice within the frame of the International Mouse Phenotyping Consortium [140] clearly demonstrates that the mitochondrial protein import machinery has an essential vital function, as the homozygous inactivation of numerous genes coding for constituents of the TOM/TIM complexes results in embryonic lethality. Over the last years, it has become clear that the phospholipid composition of the OMM and IMM plays a pivotal role in the assembly and function of the mitochondrial protein import machinery.

### 4.1. Cardiolipin

The signature phospholipid of the mitochondrial membranes is cardiolipin, as alluded to above in detail (Section 2.5). Several studies in ischemia and reperfusion injury in the heart demonstrated that the cardiolipin content is reduced by up to 25% resulting in reduced ETC assembly and activity [141,142]. Since the unsaturated fatty acids within cardiolipin consist of approximately 90% of linoleic acid [142], it was hypothesized that the dietary intake of linoleic acid would improve the outcome in heart failure patients. Indeed, experimental studies in mice revealed that linoleic acid supplementation increased the content of cardiolipin in the heart of mice and improved fractional shortening. However, the dysregulated respirasome assembly, which occurred in heart failure, could not be repaired by increased intake of linoleic acid and, thus, enhanced cardiolipin content [143]. Therefore, further studies are needed to understand which other factors are required besides the cardiolipin content in protecting the heart. Of note, cardiolipin consists of numerous unsaturated fatty acids; thus, it is tempting to speculate that the increase in reactive oxygen species in ischemia and reperfusion injury in the heart leads to oxidation of cardiolipin [144] and, thus, to dysregulated assembly not only of the ETC complexes but also of the protein import machinery.

### 4.2. Mitochondrial Telomerase Reverse Transcriptase

Recently a protein known to be imported into the mitochondria via the TOM and TIM machinery containing an *N*-terminal mitochondrial import sequence has been demonstrated to be protective in ischemia and reperfusion injury—Telomerase Reverse Transcriptase (TERT).

Telomerase has been described as being essential to counteract telomere erosion in rapidly dividing cells, such as germ cells and stem cells. However, later on, it was revealed that its catalytic subunit TERT also plays a crucial role in tissues with low proliferative capacity, such as the heart and the vasculature. In that context, it was shown that physical exercise upregulates TERT expression in the myocardium, where telomere length was not affected [145], and in the thoracic aorta [146]. Moreover, it was demonstrated that TERT is expressed in different cell types of the heart, namely in cardiomyocytes as well as in endothelial and mesenchymal cells and that it is upregulated after cardiac injury [147].

**Figure 4:**
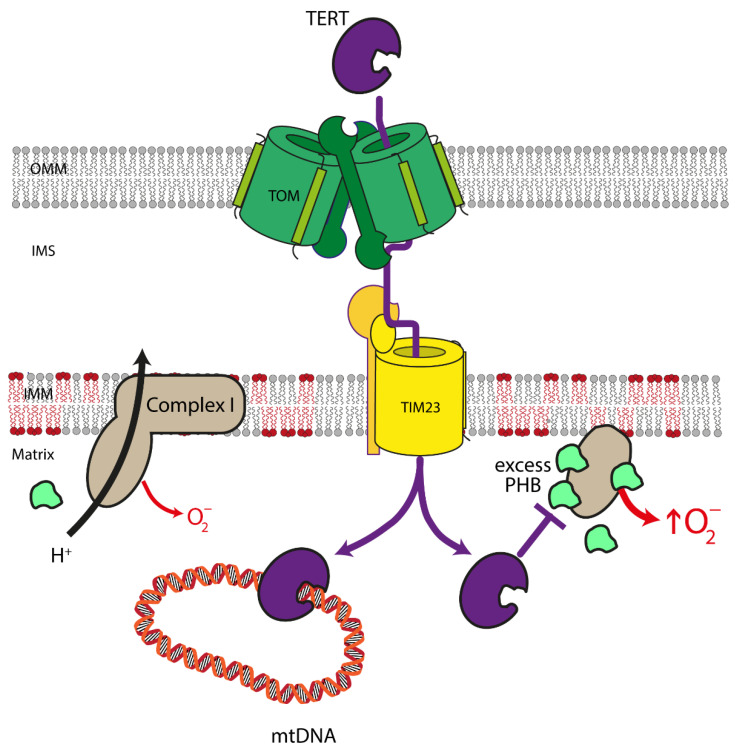
Mitochondrial functions of TERT in the cardiovascular system. In the mitochondria, Telomerase Reverse Transcriptase (TERT) binds to mitochondrial DNA (mtDNA) and protects it against damage. In addition, mitochondrial TERT improves the stoichiometry of the different subunits of complex I of the electron transport chain. Moreover, an increase in mitochondrial TERT reduces the levels of Prohibitin (PHB) in these organelles, which—when in excess—stabilizes free matrix arm subunits of complex I of the electron transport chain resulting in increased production of mitochondrial superoxide. Consequently, mitochondrial TERT improves complex I composition and activity, decreases mitochondrial reactive oxygen levels, and, thereby, contributes to enhanced mitochondrial functionality.

Notably, TERT is not only present in cell nuclei but also in mitochondria, which is easily explained by the presence of a bona fide mitochondrial targeting sequence at the N-terminus of the protein [148] in addition to the nuclear localization signal in its C-terminus. It has been demonstrated that TERT is imported into the mitochondria via the TOMM and TIMM machinery in the cardiovascular system (Figure 4) [149]. Mitochondrial TERT has protective functions, which are exerted by—among others—reduction of mitochondrial reactive oxygen species and protection of mitochondrial DNA [149,150,151,152].

Early experiments aimed at elucidating the functions of TERT in the cardiovascular system demonstrated that overexpression of TERT inhibits cardiomyocyte apoptosis ex vivo and in vivo [153] and enhances the regenerative properties of endothelial progenitor cells [154]. However, it was not clear whether nuclear or mitochondrial TERT accounts for these effects. With respect to mitochondrial functions, it was later shown that TERT-deficiency results in reduced electron transport chain activity in the heart and an increase in lesions in mitochondrial DNA [149,155], strongly supporting a mitochondrial function of TERT in this organ. Interestingly, short-term, mild oxidative stress leads to the nuclear export of TERT with a concomitant increase of its levels outside the nucleus [156], possibly in the mitochondria as a mechanism to preserve their functionality. However, prolonged exposure of endothelial cells to ROS or to high doses thereof, which results in mitochondrial dysfunction, induces TERT loss in these organelles. Intriguingly, this downregulation and nuclear export of TERT are both mediated by Src kinase, which—like other kinases and phosphatases regulating TERT—is also present in mitochondria [157]. Although mitochondrial localization of TERT and pathways regulating its levels in these organelles have also been described in the cardiovascular system, it was for the longest time impossible to distinguish between nuclear and mitochondrial functions of TERT. The analysis of mitochondrial functions in TERT-deficient animals, as well as the overexpression of nuclear or mitochondrially targeted TERT, can only provide circumstantial evidence for the functions in the two compartments, as they rely either on the complete absence of the protein or are performed on a background of endogenous TERT, which is present in both organelles.

The first study unequivocally differentiating between nuclear and mitochondrial functions of TERT in vivo and ex vivo made use of unique new mouse models containing TERT in all organs exclusively in either of the two cellular compartments [158,159]. With these mice, it was shown that only mitochondrial, but not nuclear TERT, protects against the adverse outcomes after ischemia and reperfusion injury without affecting telomere length. Animals containing TERT only in the mitochondria (mitoTERT mice) have reduced infarct and scar sizes, show improvements in vascularization in the border zone, the region adjacent to the infarct, and in cardiac functions. On the cellular level, the protective effects of mitochondrial TERT are not restricted to cardiomyocytes, which are protected against apoptosis by mitochondrial TERT but also affect the other parental cell types in the heart, namely endothelial cells and cardiac fibroblasts. Mitochondrial TERT improves the migratory capacity of endothelial cells, which is required for enhanced vascularization in the infarct area. Furthermore, mitochondrial TERT improves the differentiation of cardiac fibroblasts into myofibroblasts, a mechanically strong cell type that can passively participate in heart contractions and is necessary to replace dead cardiomyocytes in the early phase after an infarction to form a stable scar. Mechanistically, mitochondrial TERT reduces the amount of mitochondrial Prohibitin, which—when in excess—stabilizes free matrix arm subunits of complex I of the mitochondrial respiratory chain, resulting in increased superoxide production. This reduction in Prohibitin levels, together with an improved stoichiometry between the different subunits of complex I, also explains the enhanced complex I activity in heart mitochondria from the mitoTERT animals [159].

This clear demonstration of protective functions of mitochondrial TERT in myocardial infarction suggests that a therapeutic increase in mitochondrial TERT would be beneficial in cardiovascular diseases. The Telomerase activator TA-65 has already been shown to be safe for humans [160], and it has been demonstrated ex vivo that treatment with this compound leads to increased TERT levels in mitochondria as well as enhanced endothelial cell migration and myofibroblast differentiation of cardiac fibroblasts [159]. TA-65 is currently tested in a clinical trial in patients with acute coronary syndrome [161], which will potentially reveal protective effects in humans.

## 5. Conclusions

In the past years, an understanding of the biochemical processes underlying the mitochondrial preprotein import has progressed substantially, in particular also in mammalian cells. As we show above, dysfunction of mitochondrial preprotein import is correlated with or may result in severe neurodegenerative and cardiovascular pathologies. These observations corroborate the important role mitochondria play in the general maintenance of cellular function. Although mitochondrial preprotein import as such is essential for the survival of any cell, past experiments have indicated several different biochemical mechanisms how import defects contribute to disease—from a simple lack of necessary enzymatic components and the subsequent failure to provide energy to the cell up to the import system as a sensor for underlying mitochondrial dysfunction. Unfortunately, the number of corresponding experiments still remains limited, and there are significant contradictions among published ones. Future experimental studies are required to establish a more detailed and reliable mechanistic understanding of how defective mitochondrial protein import is involved in the respective diseases. In addition, these new insights have to be translated into novel therapeutic approaches to develop respective pharmacological treatments. Although many different strategies and drugs to target mitochondria have been suggested, for example, for Alzheimer’s disease, therapy efficient treatments are not available to date [90]. In particular, there are no drugs on the market directly targeting the import machinery, so more thorough fundamental studies are urgently required for the progress in this field. Moreover, one has to keep in mind that new mouse models are needed to understand the import machinery in age-related neurodegenerative and cardiovascular diseases.
